# Minimally invasive endodontics: a new era for pulpotomy in mature permanent teeth

**DOI:** 10.1038/s41415-022-5316-1

**Published:** 2022-12-16

**Authors:** Nebu Philip, Bharat Suneja

**Affiliations:** 4141510593001 College of Dental Medicine, QU Health, Qatar University, Doha, Qatar; 4141510593002 Baba Jaswant Singh Dental College and Hospital, Ludhiana, India

## Abstract

Emerging clinical and histologic evidence is challenging the long-established dogma that root canal treatment (RCTx) is the only therapeutic option for preservation of vital mature permanent teeth diagnosed with irreversible pulpitis or carious pulp exposure. Vital pulp therapy procedures like pulpotomy are not only technically simpler and more economical, but also afford patients a host of other benefits over conventional RCTx. This narrative review provides an update on the contemporary understanding of pulp pathophysiology and defence mechanisms, the proposed new diagnostic terminologies for pulpal inflammation, and how the biological characteristics of hydrophilic calcium silicate cements have enabled consistent successful outcomes for pulpotomy-treated mature teeth. The paper also details the evidence base from clinical trials and systematic reviews conducted over the past decade and outlines the practical treatment considerations for pulpotomy in mature permanent teeth.

**Supplementary Information:**

Zusatzmaterial online: Zu diesem Beitrag sind unter 10.1038/s41415-022-5316-1 für autorisierte Leser zusätzliche Dateien abrufbar.

## Key points

- Provides a contemporary update on pulp pathophysiology and defence mechanisms.
- Presents proposed new diagnostic terminologies for pulpitis.
- Details the most recent evidence base for pulpotomy in mature permanent teeth.
- Outlines treatment considerations for pulpotomy in mature permanent teeth.

**Supplementary Information:**

Zusatzmaterial online: Zu diesem Beitrag sind unter 10.1038/s41415-022-5316-1 für autorisierte Leser zusätzliche Dateien abrufbar.

## Introduction: a new era for minimally invasive endodontics

Dogmas in medicine and dentistry are often cherished with implicit faith, despite the lack of high-quality evidence. Paradigm shifts from existing treatment practices often generate great resistance, even at the risk of delivering poor-quality care to patients. One such closely held belief is that a vital mature permanent tooth diagnosed with irreversible pulpitis will require root canal treatment (RCTx) for long-term preservation of the tooth. Less invasive vital pulp therapy (VPT) procedures like pulpotomy were restricted to immature permanent teeth, with the goal of ensuring completion of their root formation (apexogenesis). However, there is now growing evidence to suggest that irrespective of whether the permanent teeth are mature or immature, if the pulpal infection and inflammation can be controlled, even ‘irreversibly' inflamed pulp tissue appear capable of healing, thus allowing for the conservative management of such teeth.^1^^,^^2^ Recent position statements from the American Association of Endodontists and the European Society of Endodontology (ESE) have concluded that ‘pre-treatment diagnosis of irreversible pulpitis is not necessarily an indication for pulpectomy',^3^^,^^4^ heralding a new era for minimally invasive VPT in mature permanent teeth. This paradigm shift suggests the need for dentists to consider offering pulpotomy as a definitive treatment modality for managing mature permanent teeth diagnosed with irreversible pulpitis or carious pulp exposures. The rationale, evidence base and treatment considerations for successful pulpotomy in vital mature permanent teeth are presented in this paper.

## Pulp defence mechanisms

Historically, the dental pulp was believed to be very vulnerable to tissue insult from bacterial carious attack and the resulting inflammation. The low compliance dentinal walls and lack of collateral circulation was thought to limit the ability of pulp tissue to accommodate increases in intra-pulpal pressure or effectively deliver humoral and cellular immune components to the injured site. The diagnostic consensus was that cariously exposed pulp in mature permanent teeth should be considered irreversibly inflamed based on the rationale that the underlying inflammation has spread throughout the pulp tissue and the restricted blood supply through the closed apices of mature teeth would not be enough to promote healing, even if the tissue insult is removed.^2^ However, studies have shown that dental pulp can not only accommodate moderate increases in intra-pulpal pressure during inflammation,^5^^,^^6^ but that the dental pulp also has an effective immune defence response.^7^^,^^8^^,^^9^

Contemporary understanding of dental pulp pathophysiology and defence mechanisms have confirmed early studies that showed the innate ability of pulp tissue to heal itself if the insult is removed.^10^ The abundant fibroblast cells in the pulpal tissue are the only non-immune cells in the body capable of activating the complement system and play a central role in modulating the repair and healing potential of pulp.^11^ Besides pulp fibroblasts, adult dental pulp stem cells also contribute to the regenerative potential of pulp in mature permanent teeth. Recent data have suggested that pulp defence mechanisms are mediated via the following pathways: i) complement activation by pulp fibroblasts expresses significant anti-inflammatory potential and also contributes to tissue regeneration by recruiting pulp progenitors;^12^ ii) pulp fibroblasts can directly induce lysis of cariogenic bacteria;^13^ iii) chemokines released from injured pulp tissue attract mesenchymal dental pulp stem cells that can differentiate into odontoblast-like cells and induce reparative dentine formation;^14^ and iv) synthesis and release of antimicrobial peptides by dental pulp stem cells.^9^

Histopathologic and histobacteriologic studies have shown that, in teeth with irreversible pulpitis or carious pulp exposures, there is a bacterially colonised necrotic area of varying dimensions in the pulp chamber.^15^ However, few millimetres away from the bacterially colonised necrotic tissue, it is not unusual to find the healthy pulpal architecture that is generally free from inflammation and bacteria.^16^^,^^17^ Innate and adaptive immune defence mechanisms equip the pulp to limit the spread of bacterial infection.^18^^,^^19^ If no treatment is rendered to eliminate the infected pulp, the pulp infection at the carious exposure site will gradually spread to involve the entire coronal pulp, although the radicular pulp can still remain free from infection.^15^ In theory, if the infected coronal pulp is completely removed, a favourable environment can be created for radicular pulpal healing as the immunoinflammatory cells get eliminated by apoptosis and the odontoblast-like cells induce dentine bridge formation. Taken together, the histological picture of a severely inflamed pulp may not always be a sign of irreversibility in terms of infection.

Thus, the current interpretation of pulp inflammation includes the understanding that ‘irreversible' pulpitis need not to be seen as a one-way route towards pulp cell impairment and subsequent necrosis, but as a ‘double-edged sword', where a so-called wanted inflammation, given the right balance, can result in pulpal repair and healing. On the other hand, if the pulpal inflammation is sustained and uncontrolled, it will inevitably lead to an infected pulp cavity and tissue necrosis.^20^^,^^21^ However, the demarcation point at which pulpal inflammation becomes truly irreversible is difficult to determine based solely on patient symptoms and currently available diagnostic tests.^2^

## New diagnostic terminology for pulpitis

Traditionally, identifying reversible/irreversible pulpitis relied on a patient's subjective description of symptoms and pulp sensibility tests. However, the simple dichotomous way of describing inflamed vital pulp as reversible or irreversible pulpitis does not match the current understanding of pulp biology and the defensive response of the pulp complex.^22^ With histologic evidence showing that there is no discrete boundary that would render a pulp irreversibly inflamed and beyond repair, it may be better to consider pulpitis as a temporally and spatially graded disease.^3^ The contemporary understanding of pulpal inflammation and healing have led to calls to revise the existing diagnostic nomenclature.^23^^,^^24^ Wolters and co-workers expanded the classification of pulpitis based on patient symptoms and possible histologic picture and related them to different VPT modalities (Table 1).^24^ The ESE proposed the term ‘partial irreversible pulpitis' as possibly a more accurate clinical reflection of the histological picture,^4^ while others have suggested that the diagnostic term for pulpal inflammation should be confined to ‘pulpitis' without any further designation.^2^ In the context of practising minimally invasive endodontics, the terms ‘reversible' and ‘irreversible' are considered obsolete, especially considering our improved understanding of the pulp biology and the importance of preserving vital pulp.^25^ The proposed new diagnostic terminologies can guide clinicians in choosing more conservative therapeutic options when treating patients with caries-induced pulpal inflammation.

**Table 1:** Proposed diagnostic classification of inflamed vital pulp and suggested treatment options^24^

**Pulp status**	**Clinical symptoms**	**Histological picture**	**Treatment suggested**
Initial pulpitis	Heightened but non-lingering response to thermal tests No spontaneous pain or percussion sensitivity	Limited local inflammation confined to coronal pulp	Indirect pulp capping
Mild pulpitis	Heightened lingering response to thermal tests lasting up to 20 seconds No spontaneous pain but possible percussion sensitivity	Limited local inflammation confined to coronal pulp	Indirect pulp capping
Moderate pulpitis	Strong, heightened and lingering response to thermal tests which can last for minutes Spontaneous dull pain that is controlled with analgesics Possibly percussion sensitive	Extensive local inflammation confined to coronal pulp	Partial/full coronal pulpotomy
Severe pulpitis	Clear pain reaction to thermal stimuli Severe spontaneous sharp or dull pain with limited relief from analgesics Very sensitive to percussion	Extensive local inflammation of coronal pulp possibly extending into root canals	Full coronal pulpotomy if haemostasis can be achieved. If bleeding from pulp stumps persists, more inflamed tissue is removed from canals. If bleeding still persists, full pulpectomy is done

## Why pulpotomy in mature permanent teeth?

Full pulpectomy and RCTx of vital mature permanent teeth with irreversible pulpitis or carious pulp exposure can be considered as a prophylactic procedure to prevent further pulpal infection and subsequent development of apical periodontitis (AP).^1^ There is no doubt that a correctly performed RCTx can achieve high success rates.^26^^,^^27^ Unfortunately, cross-sectional studies from across the world have shown that up to 40% of root filled teeth are technically inadequate with persistent AP.^28^^,^^29^^,^^30^^,^^31^^,^^32^ Managing irreversible pulpitis in mature permanent teeth with pulpotomy could potentially have a number of advantages over conventional RCTx: i) treatment procedure is technically less challenging, avoiding the complications associated with difficult root canal anatomy; ii) it preserves the proprioceptive sensation of the tooth; iii) biological immune response from the retained pulp tissue can prevent infection of the apical area; iv) regenerative and repair potential of the pulp is retained; v) structural integrity of the tooth is maintained, lowering the risk of fracture; vi) there is significant reduction in pain and discomfort to the patient; and vii) it saves time and cost for both the patient and public health systems.

A potential concern after full pulpotomy in mature permanent teeth is the occurrence of pulp canal obliteration leading to AP. However, the development of AP in pulpotomised permanent teeth is a sequela of pulp infection, either due to coronal restoration microleakage or incomplete pulp disinfection during the pulpotomy procedure, and not due to the pulp canal obliteration itself.^1^ Canal calcification in pulpotomised teeth without pulp infection will not lead to AP and further treatment intervention should not be required.^33^

## Contemporary pulpotomy medicaments

The pulpotomy medicament to be placed directly over the remaining pulp tissue should ideally be able to provide a good seal against long-term bacterial leakage, stimulate healing and repair of the remnant pulp tissue, and promote dentinogenesis.^34^ Calcium hydroxide (CH) was among the earliest and most popular medicaments used for VPT based on its high alkalinity and ability to stimulate reparative dentine formation. However, CH also induced several healing complications when placed directly over vital pulp, with studies showing the success rates of CH VPT significantly declining over time.^35^ The drawbacks of using CH for VPT included: i) tunnel defects in the newly formed dentine resulting in an ineffective seal; ii) high solubility of CH in oral fluids; and iii) poor adhesion to pulp floor due to its hydrophobicity. These healing complications could be the reason why CH demonstrated a lower range of clinical success (34-92%) when used as the pulpotomy medicament in mature permanent teeth.^36^^,^^37^^,^^38^^,^^39^ Despite its lower costs, the use of CH as a pulpotomy medicament in mature teeth can no longer be recommended.

Recent decades have seen the development of bioactive hydrophilic calcium silicate cements (CSCs), such as mineral trioxide aggregate, calcium-enriched mixture, Biodentine, and bioceramics for use in VPT procedures. These hydrophilic CSCs have demonstrated more consistent clinical success (85-100%) when used as the pulpotomy medicament in mature permanent teeth.^40^^,^^41^^,^^42^^,^^43^^,^^44^^,^^45^^,^^46^^,^^47^ The contrast in clinical outcomes was especially stark when direct comparisons were made between CH and CSCs for VPT.^39^^,^^48^^,^^49^ New-generation bioactive CSCs are not only dimensionally stable with excellent sealing abilities, but also have beneficial biocompatible, immunomodulatory and osteogenic properties.^50^^,^^51^ Recent studies have shown that CSCs can induce the release of regenerative dentine-bound growth factors, upregulate angiogenesis, and stimulate cellular differentiation of dentine-forming cells.^52^^,^^53^ These biological properties provide for better pulpal healing and improved quality of the mineralised dentine bridge over the pulp, contributing towards successful pulpotomy outcomes even in mature permanent teeth.

## State of evidence

Pulpotomy has traditionally not been part of the treatment considerations for mature permanent teeth diagnosed with irreversible pulpitis. However, there is now increasing evidence from retrospective studies,^54^^,^^55^^,^^56^ prospective cohort studies^40^^,^^41^^,^^47^^,^^57^^,^^58^^,^^59^^,^^60^ and randomised controlled trials,^39^^,^^42^^,^^43^^,^^44^^,^^45^^,^^46^^,^^61^^,^^62^^,^^63^^,^^64^^,^^65^^,^^66^^,^^67^^,^^68^^,^^69^^,^^70^ showing high success rates for pulpotomy in treating mature permanent teeth with irreversible pulpitis or carious pulp exposures (online Supplementary Table 1). Systematic reviews, meta-analyses and recent umbrella reviews (Table 2) have also concluded that pulpotomy could be a prospective substitute to conventional RCTx in managing vital mature permanent teeth diagnosed with irreversible pulpitis or carious pulp exposure.^71^^,^^72^^,^^73^^,^^74^^,^^75^^,^^76^

**Table 2:** Systematic/umbrella reviews on pulpotomy in mature permanent teeth (2016-2021)

**Study design**	**Authors/year**	**Population**	**Intervention**	**Studies included**	**Conclusions**
SR and MA	Alqaderi *et al*.^71^2016	Vital mature posterior teeth with carious pulp exposure	FP	6	FP has favourable success rates in treating carious pulp exposure of vital mature permanent teeth
SR and MA	Li *et al*.^74^2019	Cariously exposed vital mature posterior teeth including those with IP	FP	21 for SR and 5 for MA	FP is a prospective substitute for RCTx in managing permanent teeth with carious pulp exposures, even with IP
SR	Cushley *et al*.^72^2019	Mature posterior teeth with symptomatic IP	FP	8	High success for FP in treating teeth with IP
SR and MA	Elmsmari *et al*.^76^2019	Vital mature posterior teeth with carious pulp exposure	PP	11	PP has high success rates in treating cariously exposed permanent posterior teeth up to two years
SR	Santos *et al*.^75^2021	Mature posterior teeth with symptomatic IP	FP and PP	12	FP and PP performed with CSCs had favourable outcomes in mature posterior teeth diagnosed with IP
UR	Leong and Yap^73^2021	Vital mature posterior teeth with carious pulp exposure	DPC, PP, FP	6	PP and FP had higher and more predictable success rates than DPC and could be considered as an alternative to RCTx
Key: SR = systematic review MA = meta-analysis UR = umbrella review IP = irreversible pulpitis FP = full pulpotomy PP = partial pulpotomy DPC = direct pulp capping RCTx = root canal treatment CSC = calcium silicate cements					

## Treatment considerations for pulpotomy in mature teeth

While the pulpotomy procedure is technically less challenging than conventional RCTx, it still requires strict adherence to procedural guidelines to achieve long-term success. Parameters like correct diagnosis of initial pulp status; strict aseptic operative technique; disinfection and haemostasis of remanent pulp; use of bioactive hydrophilic pulpotomy medicaments; and provision of immediate definitive coronal restorations will influence pulpotomy outcomes in mature permanent teeth. Treatment considerations for pulpotomy in mature permanent teeth, based on a synthesis of evidence from successful clinical studies, are detailed below.

### Diagnosis: symptoms, sensibility tests and radiographs

**Fig. 1:**
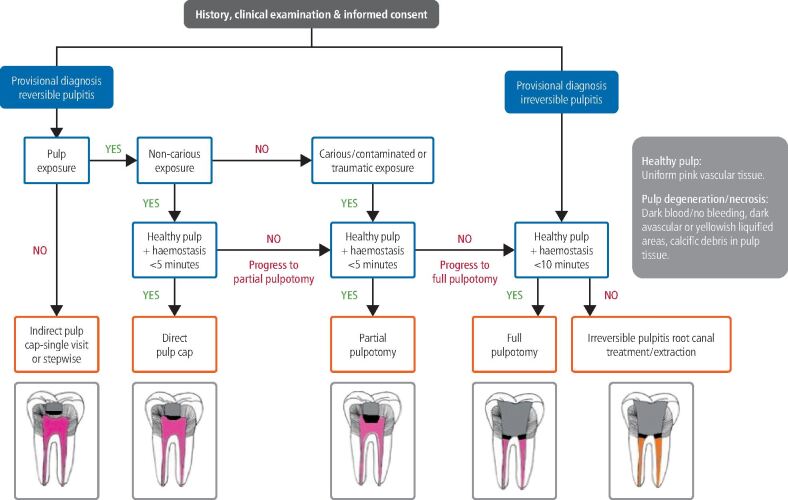
Decision tree for inflamed vital pulp in mature permanent teeth. Reproduced with permission from Yong *et al.,* ‘Conservative pulp therapy in the management of reversible and irreversible pulpitis', *Australian Dental Journal*, 2021, Australian Dental Association^77^

Despite its limitations, pre-operative diagnosis of pulpitis based on clinical signs and symptoms and response to pulp sensibility tests can serve as an initial guide in choosing the best therapeutic option for mature permanent teeth with pulpal inflammation (see decision tree in Fig. 1).^77^ The radical change in the available treatment options is that full pulpotomy is now indicated even for mature teeth with symptoms typical of irreversible pulpitis (severe spontaneous or continuous pain with exaggerated lingering responses to sensibility tests). Furthermore, full pulpotomy can also be performed in vital mature teeth with signs of AP (pain on percussion) or with periapical lesions on the radiograph. Carious pulp exposures in vital mature teeth without signs and symptoms of irreversible pulpitis or AP can initially be treated even more conservatively with partial pulpotomy, progressing to full pulpotomy if haemostasis is not achieved. However, VPT is contraindicated in mature teeth diagnosed with pulpal necrosis (confirmed by negative response to sensibility tests or intra-operatively by the lack of pulpal bleeding).

### Aseptic operative technique

Successful outcomes for pulpotomy are contingent on strict adherence to an aseptic operative technique. These measures include: i) mandatory rubber dam isolation; ii) pre-operative crown disinfection before caries excavation with 2% chlorhexidine (CHX) or 5% sodium hypochlorite (NaOCl); iii) minimising further bacterial contamination of pulp by the removal of all carious tissues, starting at the periphery of the cavity and then progressively over the pulp chamber roof; and iv) mandatory use of a fresh sterile bur (different from the caries excavation bur) when de-roofing the pulp chamber.

### Pulp amputation and haemostasis

Pulpotomy outcomes will depend on the severity of pulp inflammation and ability to obtain haemostasis after the removal of inflamed tissue. Once the pulp is exposed, flushing the cavity with CHX or NaOCl can minimise the bacterial load and prevent lodgement of dentinal debris into pulpal tissue. Pulp amputation should be carried out with sterile high-speed rotatory bur under copious water irrigation. Another critical step after pulp exposure is the intra-operative assessment of pulp vitality. Direct visualisation of pulp tissue (preferably under magnification) during and after haemostasis not only provides additional diagnostic information about degree of pulp inflammation, but can also help identify potential necrotic tissues that require removal before application of the pulpotomy medicament.^3^ Healthy vital pulp will present as uniformly red vascular tissue, while non-vital necrotic pulp presents as dark avascular tissue with minimal bleeding or as yellowish liquefied areas or with calcific debris embedded in the pulp tissue.^77^

Haemostasis and disinfection of the resected pulp tissue is achieved either by placement of a NaOCl-soaked sterile cotton pellet over the amputated pulp or by passive NaOCl irrigation. NaOCl in concentrations ranging from 0.5-5% can be used in direct contact with pulpal tissues without compromising pulp cell recruitment, cytodifferentiation, and reparative dentine formation.^34^^,^^78^^,^^79^ Besides haemostatic effects, NaOCl also disinfects the dentine-pulp interface and removes adherent biofilms.^78^ Although physiologic saline has been used in place of NaOCl for haemostasis, it lacks disinfection properties, possibly resulting in poorer outcomes when compared with NaOCl haemostasis.^80^ The use of more effective haemostatic agents (for example, ferric sulphate or hydrogen peroxide) should be avoided as they tend to mask the true inflammatory status of the pulp.^4^

The time taken to achieve haemostasis after pulp amputation has been used as an indicator for the degree of pulpal inflammation and as a prognostic factor for procedural success of VPT.^81^ However, a retrospective study that investigated the ‘time to stop bleeding' after pulpotomy in vital mature teeth with carious pulp exposures concluded that bleeding time had no effect on treatment outcomes^55^ and clinical studies have reported successful outcomes for bleeding times ranging from 1-25 minutes.^79^ Recent reviews suggest that bleeding duration may not be a true indicator of pulpal inflammatory status^82^^,^^83^ and therefore achieving immediate haemostasis need not be a determining factor for successful pulpotomy outcomes. Nevertheless, persistent bleeding beyond ten minutes, despite attempts at haemostasis, should be considered as a contraindication for pulpotomy in mature permanent teeth and RCTx or extraction should be preferred in these cases.^2^

### Pulpotomy medicament and coronal restoration

Probably the most critical factor in achieving favourable pulpotomy outcomes is adequate sealing of the remnant pulp tissue with the bioactive medicament and a definitive coronal restoration. Once haemostasis is achieved, 2-3 mm of a hydrophilic CSC should be directly adapted over the pulp stumps, ensuring that there is no porosity or excess cement on the pulp chamber walls. Immediate placement of a definitive coronal restoration is also recommended to prevent microleakage, protect the bioactive medicament, reduce post-operative sensitivity, and establish foundation for future cuspal coverage restoration, should it be required.^3^ The data on placing full coverage crowns on pulpotomy-treated teeth are limited, with a couple of studies reporting that placing crowns on such teeth had higher success rates compared to resin composite or amalgam restorations.^54^^,^^60^ In addition, 100% success rates have been demonstrated following placement of stainless steel crowns in pulpotomised permanent molars of children.^47^ A 3-6 month waiting period has been suggested before additional tooth preparation for cuspal coverage, as early endodontic failures tend to occur within this period.^59^ If clinical and radiographic outcomes of the pulpotomy treatment are successful after this waiting period, a full coverage restoration should be strongly considered for long-term survival of the pulpotomised tooth.^3^^,^^77^

### Follow-up and prognosis

The ESE recommends that teeth that receive VPT should be assessed with clinical, radiographic and sensibility testing at 6 and 12 months post-operatively, and thereafter at yearly intervals for up to four years.^4^ The clinical outcome measures for success are an asymptomatic functional tooth, no tenderness to percussion or palpation, and no swelling or sinus tract associated with the treated tooth. Radiographically, there should be no signs of internal root resorption, evident healing of any pre-operative periapical lesions, and no new periapical pathologies. Sensibility tests should elicit a normal response in teeth that receive pulp capping or partial pulpotomy. However, teeth that have undergone full pulpotomy will not be responsive to sensibility tests and in these cases, radicular pulp is considered normal unless there are clinical or radiographic signs of failure.^2^

Recent clinical trials suggest that early failures of pulpotomy-treated mature teeth (that is, those that fail within 3-6 months of treatment) are mostly due to endodontic causes (for example, inaccurate assessment of inflammatory status of pulp), while later failures tend to reflect restorative causes (for example, pulp space reinfection due to poorly sealed coronal restorations).^59^^,^^60^ Clinical trials have shown that age, sex, previous restorations, site of carious exposure, and presence of pre-operative periapical lesions do not appear to be significant factors in deciding prognosis of pulpotomy-treated mature teeth.^42^^,^^58^^,^^59^ The only potential prognostic predictive factors found in clinical studies of pulpotomy in mature teeth were pre-operative pain (for early failures) and the type of definitive coronal restoration used (for late failures).^59^^,^^60^

## Conclusions

The drive to practise minimally invasive endodontics, improved understanding of dentine-pulp defence mechanisms, introduction of bioactive pulp medicaments, and accumulating evidence from clinical and radiographic outcome studies have resulted in pulpotomy being increasingly considered as a therapeutic alternative to traditional RCTx, even in mature teeth. Pulpotomy could be an especially attractive option for patients who do not have access to specialist endodontic care or cannot afford its costs. The ESE position statement, while cautiously recommending such treatments, suggests the need for more robust long-term evidence before pulpotomy can be routinely recommended as a substitute for RCTx.^4^ Attempts to collate further high-quality clinical evidence on the long-term effectiveness of pulpotomy in treating mature teeth diagnosed with irreversible pulpitis are currently underway around the world (Table 3). Within the UK, three clinical trials on pulpotomy in permanent teeth are in progress, including the National Institute of Health Research-funded multi-centre primary care PIP trial and the Northern Ireland Public Health Agency funded REFORM trial. The day is probably not too far when pulpotomy can be routinely offered as the first line of treatment for vital mature permanent teeth diagnosed with irreversible pulpitis.

**Table 3:** Ongoing clinical trials investigating pulpotomy in mature teeth with symptomatic irreversible pulpitis

**Study design**	**Study title**	**Estimated enrolment**	**Estimated completion**	**Trial registry**	**Trial identifier and location**
RCT	Success and quality of life following complete pulpotomy and root canal treatment in teeth with clinical signs indicative of irreversible pulpitis	100	August 2022	ClinicalTrials.gov	NCT05190406 India
Multi-centre RCT	Pulpotomy vs. root canal treatment in managing irreversible pulpitis	168	April 2023	ClinicalTrials.gov	NCT03956199 U.K
RCT	Full pulpotomy vs. partial pulpotomy in the management of teeth with clinical diagnosis of irreversible pulpits: a randomised clinical trial	200	January 2024	ClinicalTrials.gov	NCT05279820 Jordan
RCT	Quality of life, satisfaction and outcome after full pulpotomy compared to root canal therapy	60	January 2024	ClinicalTrials.gov	NCT05279781 Jordan
Multi-centre RCT	Traditional or minimally invasive endodontics for managing carious teeth with symptomatic pulpitis - a pragmatic randomised trial in general dental practice in Northern Ireland (REFORM)	164	April 2024	ISRCTN	49302282 U.K
Multi-centre non-randomised	Pulpotomy for the management of irreversible pulpitis in mature teeth - (PIP trial) feasibility study	40	June 2024	ISRCTN	17973604 U.K
RCT	Comparative effectiveness of VPT vs. RCTx in the management of irreversible pulpitis	120	March 2028	ClinicalTrials.gov	NCT04922229 U.S.A
Key: RCT = randomised controlled trial VPT = vital pulp therapy RCTx = root canal treatment ISRCTN = international standard randomised controlled trial number					

### Supplementary Information

Supplementary Table 1 (PDF 86KB)
