# Supplementary material for: Minimally invasive endodontics: a new era for pulpotomy in mature permanent teeth
Source: Br Dent J. 2022 Dec 16;233(12):1035–41. doi: 10.1038/s41415-022-5316-1 (PMC9758046; doi:10.1038/s41415-022-5316-1)
Supplement: Supplementary file 1 — Supplementary Table 1 (PDF 86KB) [file 41415_2022_5316_MOESM1_ESM.pdf]

Supplementary Table 1 Clinical studies investigating VPT in permanent teeth (2010-2022)

Study design	***Authors, year and country***	***Baseline sample size and age***	***Teeth type and initial diagnosis***	***VPT***	***PM***	***Primary outcome***	***Follow-up period***	***Results***
RCT	Asgary and Eghbal[61] 2010 Iran	407 teeth 9–65 years	Mature molars. IP	FP	CEM	Post-treatment pain	7 days	FP had better pain reducing effects than RCTx
RCT	Asgary *et al.*[63] 2013 Iran	407 teeth 9–65 years	Mature molars. IP	FP	CEM	Clinical and radiographic success	1 year	FP (97.6%) and RCTx (98.3%) had similar clinical success rates. Radiographic success was significantly better for FP (92.8%) than RCTx (81.2%)
Prosp.	Alqaderi *et al.*[67] 2014 Kuwait	29 teeth 10–15 years	Mature premolars and molars. Deep caries, RP	FP	MTA	Clinical and radiographic success	47 months	Overall success rate of FP was 90%
RCT	Asgary *et al.*[62] 2014 Iran	407 teeth 9–65 years	Mature molars. IP	FP	CEM	Clinical and radiographic success; Treatment time/cost	2 years	FP and RCTx had similar clinical and radiographic success rates; Treatment time/costs were significantly better for FP
RCT	Asgary *et al.*[42] 2015 Iran	407 teeth 9–65 years	Mature molars. IP	FP	CEM	Clinical and radiographic success	5 years	No significant difference in the five-year success rates of FP and RCTx
Retro.	Kunert *et al.*[54] 2015 Brazil	273 teeth 8–79 years	Not reported	FP	CH	Clinical and radiographic success	1–29 years	FP success rates of 89% at 1 year and 63% at 10 years
RCT	Kumar *et al.*[66] 2016 India	54 teeth 14–32 years	Mature lower molars. IP	FP	MTA CH PRF	Clinical and radiographic success	1 year	FP with the three medicaments had similar clinical and radiographic success rates
Retro.	Linsuwanont *et al.*[55] 2017 Thailand	66 teeth 7–68 years	Young/mature molars. Carious exposure, IP and AP	FP	MTA	Clinical and radiographic success	62 months	Overall success rates of FP was 87.3%, including success rates of 84% and 76% for teeth with IP and AP, respectively

Prosp.	Taha *et al.*[41] 2017 Jordan	52 teeth 11–51 years	Mature molars. Carious exposure, IP and AP	FP	MTA	Clinical and radiographic success	3 years	Overall 92.7% success rate for FP after 3 years
Prosp.	Qudeimat *et al.*[47] 2017 Kuwait	23 teeth 7–13 years	Young/mature molars. IP	FP	MTA	Clinical and radiographic success	19-74 months	100% clinical and radiographic success rates
RCT	Asgary *et al.*[69] 2017 Iran	412 teeth 9–65 years	Mature molars. IP and AP	FP	MTA CEM	Clinical and radiographic success	5 years	5-year success rates of FP were similar for both MTA (85%) and CEM (78%)
RCT	Taha and Khazali[39] 2017 Jordan	50 teeth 20–52 years	Mature molars. IP	PP	MTA CH	Clinical and radiographic success	2 years	Success rates of MTA FP (85%) were significantly better than CH FP (43%)
RCT	Galani *et al.*[65] 2017 India	54 teeth 15–50 years	Mature 1st & 2nd lower molars. Carious exposure	FP	MTA	Clinical and radiographic success; post-operative pain	18 months	Overall success rates were similar for FP (85%) and RCTx (87.5%); Pain relief significantly better for FP
Prosp.	Taha and Abdelkader[40] 2018 Jordan	64 teeth 19–65 years	Mature molars. IP with or without AP	FP	Biod	Clinical and radiographic success	1 year	FP clinical and radiographic success rates were 100% and 98.4%, respectively
RCT	Asgary *et al.*[64] 2018 Iran	302 teeth 12–75 years	Mature molars. Deep caries, RP, IP, AP	IPC DPC PP FP	CEM	Clinical and radiographic success	1 year	Comparable success rates for all four VPT procedures
RCT	Awawdeh *et al.*[43] 2018 Jordan	68 teeth 16–59 years	Mature incisors, premolars, molars. Deep caries, RP	DPC PP	MTA Biod	Clinical and radiographic success	3 years	Overall success rates were similar for VPT procedures using MTA (96%) and Biod. (91.7%)
RCT	Uesrichai *et al.*[44] 2019 Thailand	69 teeth 6–18 years	Young/mature molars. IP	PP	MTA Biod	Clinical and radiographic success	14–50 months	Overall success rates were similar for PP with MTA (92%) and Biod. (87%)
Prosp.	Tan *et al.* 2020[60]	61 teeth 21–75 years	Mature teeth. Carious exposure	FP	MTA	Pulp survival rates	4 years	Overall pulp survival was 90.2%
RCT	Eghbal *et al.*[68] 2020 Iran	550 teeth 10–61 years	Mature molars. Carious exposure, IP	FP	MTA CEM	Post-operative pain relief	7 days	RCTx and FP with MTA or CEM had comparable post-operative pain relief
Prosp.	Guan *et al.*[57] 2021 China	59 teeth 6–20 years	Young/mature pre-/molars. Carious exposure, IP	DPC PP FP	iRoot	Clinical and radiographic success	6–36 months	Overall success rates of the three VPT procedures was 91.2%

Retro.	Eggman et al.[56] 2021 Switzerland	111 teeth 24–50 years	Mature premolars, molars, incisors, and canines. Carious exposure	PP	MTA	Pulp vitality and tooth survival	1–5 years	Success rate of PP in maintaining pulp vitality was 89.1%, with 98.4% tooth survival rates
Prosp.	Rechithra et al.[58] 2021 India	80 teeth 16–35 years	Mature lower molars. IP	FP	MTA	Clinical and radiographic success depending	1 year	Overall success rate for FP was 93.7%, and carious lesion site did not influence pulpotomy
RCT	Asgary et al.[45] 2021 Iran	157 teeth 10–60 years	Mature molars. Carious exposure, IP, and AP	FP	MTA CEM	Clinical and radiographic success	2 years	Clinical and radiographic success rates were similar for MTA/CEM FP and RCTx (98-100%)
RCT	Koli et al.[46] 2021 India	60 teeth 18–35 years	Mature lower molars. IP and AP	FP	MTA	Clinical and radiographic success	1 year	Clinical success rates were similar for MTA (93.3%) and RCTx (90%) with no differences in
Prosp.	Taha and Al- khatib[59] 2022 Jordan	109 teeth 14–60 years	Mature molars. Carious exposure, RP and IP	FP	MTA	Survival rate	4 years	Overall survival rate was 83.8%
RCT	Taha et al.[70] 2022 Jordan	193 teeth 10-70 years	Mature molars. IP and RP	FP	MTA Biod TF	Clinical and radiographic success	1 year	Overall success rates were similar for FP with MTA (91.8%), Biod (93.3%), and TF (91.9%)

Key:

VPT = vital pulp therapy

PM = pulpotomy medicament

RCT = randomised controlled trial

Prosp = prospective

Retro = retrospective

IP = irreversible pulpitis

RP = reversible pulpitis

AP = apical periodontitis

RCTx = root canal treatment

FP = full pulpotomy

PP = partial pulpotomy

DPC = direct pulp capping

IPC = indirect pulp capping

CEM = calcium-enriched mixture

MTA = mineral trioxide aggregate

Biod: Biodentine

CH = calcium hydroxide

PRF = platelet rich fibrin

TF = TotalFill
